# Birth Preparedness and Its Effect on Place of Delivery and Post-Natal Check-Ups in Nepal

**DOI:** 10.1371/journal.pone.0060957

**Published:** 2013-05-15

**Authors:** Dipty Nawal, Srinivas Goli

**Affiliations:** 1 Demography, International Institute for Population Sciences, Deonar, Mumbai, India; 2 Development Study, Giri Institute of Development Studies, Lucknow, India; Tehran University of Medical Sciences, Iran (Islamic Republic of)

## Abstract

**Introduction and Rationale:**

Number of demographically laggard countries will forego MDGs 4 and 5, and Nepal is not an exception to it. International reports reveal that, lack of adequate birth preparedness is one of the greatest hurdles in achievement of MDG 4 and 5. However, lack of comprehensive evidence at country level in developing countries like Nepal is a hindrance for policy making. In this context, this study estimated birth preparedness among Nepali women and its association with institutional delivery and postnatal care in Nepal.

**Methodology/Principal Fining:**

Secondary data such as latest round of Nepal Demographic and Health Survey Data (NDHS, 2011) has been used in the study. Bivariate and multivariate models are applied as the methods of data analyses. Results reveals that only 32 per cent of women in Nepal have birth preparedness. The women who are well prepared belong to higher age group (45%), higher education (36%) and with higher women autonomy (86%). Women, who are well prepared for child birth (OR = 3.137, p<0.01) have a greater likelihood of going for institutional deliveries that women with no preparation (OR = 1). However, irrespective of level of birth preparedness, women in Nepal preferred to deliver the baby in public health facility that private health Facility.

**Conclusion and Implications:**

Findings reveal that birth preparedness is one of the critical factors in determining the likelihood of having institutional delivery and checkups after delivery. At policy perspective, this study fosters that developing countries like Nepal have to ensure adequate and universal birth preparedness in order to achieve goal 4 and 5 of MDGs.

## Introduction and Rationale

During the early 1980s, a half-million women died every year during pregnancy, childbirth and postpartum period [1]. However, the recent estimation of the United Nation reveals that maternal deaths are counted fewer than 287,000 women every year [2]. Though, the maternal deaths are falling across the globe, but, relatively high maternal mortality ratio in developing countries is a cause of concern for numbers of countries worldwide [3], [4]. More, latest estimates show that maternal deaths in ten countries count for 60 per cent of the global maternal mortality burden [2]. Two regions: sub-Saharan Africa (56%) and Southern Asia (29%) together contributes to 85% of the global maternal mortality burden in 2010 [1], [5], [6], [7].

Innumerable recent literature have pointed out the lack of adequate birth preparedness as critical factors behind the sluggish progress towards the maternal target in the laggard countries [8], [9], [10], [11], [12], [13], [14]. Birth preparedness, is a process of preparing for pregnancy complications emergency obstetric care and delivery care in terms of saving money, transportation and blood arrangement [11], [12], [14], [15], [16]. Birth preparedness is also considered as an intervention for preventive behaviour and programmatic approach to other socio-economic and cultural barriers, which limit the requisite to avail the health facility [9], [12], [13], [17] especially the referral pathways [18], skilled medical practitioner [21], and adequate equipment and infrastructure [19], [24], and other requisite over the course of delivery as well as child care [20], [21], [22], [23], [24], [25], [26], [27]. Utilization of birth preparedness kits, community involvement in counselling and physiological support to child bearing, save women from maternal deaths occurred during labour pain, delivery and within the 24 hours of the post partum and other inter-correlated sign of danger [8], [19], [28], [29], [30], [31], [32], [33], [34].

### Nepal Context

In developing countries like Nepal where, maternal mortality is high due to obstacles in accessibility of health care facility and that of health personals [35], [36], [37]. Activities to improve birth preparedness and identifying complications at household and community levels are critical to improve mother's survival [7], [11]. With this identification, the minimum level of services would be mandatory for all Nepali women under the essential health care services package in Nepal [38]. Studies in Nepal have identified that the mother's consciousness and active participation in neonatal and child health is imperative [39], [40], [41].

Furthermore, studies have focussed on safe motherhood prioritised birth preparedness as a strategy to reach MDG goal 4 and 5 [42], [2]. However, at the end of 2008–2009, the birth preparedness package (BPP) had been rolled out in all 75 districts of Nepal [43]. Similarly, a maternity incentive scheme was adopted in 2005 to encourage women to use health facilities and to improve access to maternity care services [44]. In this context, it is necessary to evaluate the model that construct as an intervention (birth preparedness) to outcome (Institutional delivery and post natal care) approach. Therefore, the principal objective of this paper is to assess the birth preparedness and its association with institutional delivery and postnatal check-up in Nepal.

## Materials and Methods

### Data and Sample Size

This study has used data from the latest round of Nepal Demographic and Health Survey (NDHS-4, 2011) [43]. The survey sample was designed to yield representative information for most indicators of the country as a whole, for urban and rural areas, for the three ecological zones (mountain, hill, and terrain), and for each of the 13 domains obtained by cross-classifying the three ecological zones and the five development regions (Eastern, Central, Western, Mid-western, and Far-western). The primary objective of the 2011 NDHS was to provide estimates with an acceptable level of precision for important population characteristics such as fertility, contraceptive prevalence, maternal and child health and mortality. The survey was designed to target a sample of 11,095 households and it was expected to interview a total of 13,200 women of age 15–49 in the sample households and all men of age 15–49 in a sub-sample of one in every two households selected for the woman's interview. Women and men were considered eligible for interview if they were usual members of the household or if they stayed in the household at the night before the survey [43].

### Sampling Design and Weights

The sample design represents the country as a whole, 13 domain obtained by cross-classifying the three ecological zone and five development region (stratum). Nepal is administratively divided into 75 districts, which are further divided into smaller administrative units known as Village Development Committees (VDCs) and Municipalities. VDCs and Municipalities have been further divided into two wards; larger ward and sub-wards. An Enumeration Area (EA) is defined as a ward in the rural areas and a sub-ward in the urban areas. Two stage stratified cluster sampling have been used in NDHS 2011. The sample is selected with unequal probability to expand the number of cases available (and hence reduce sampling variability) for certain areas or subgroups for which statistics is needed. In the first stage, probability proportionate-to-size has used for the selection of EAs. To obtain target size of each domain, the ratio of urban EAs over rural EAs is each domain was considered roughly 1 to 2. In the second stage, 35 household forms urban EA and 40 household in each rural EA were randomly selected.

Sampling weight was calculated on the basis of sampling probability separate for each sampling stage. Sampling weights are adjustment factors applied to adjust for differences in the probability of selection of cases in a sample, either due to design or happenstance. When weights are calculated because of sample design, corrections for differential response rates are also made. There are two main sampling weights in the NDHS-2011: household weights and individual weights. The household weight for a particular household is the inverse of its household selection probability multiplied by the inverse of the household response rate of its household response rate group. The individual weight of a respondent's case is the household weight multiplied by the inverse of the individual response rate of their individual response rate group [28].

### Outcome Measurements

The study measures two outcome variables namely, Place of Delivery and Post-natal Check-ups. For increasing the percentage of births delivered in health facilities it is important to reduce deaths arising from complications of pregnancy [43]. It is imperative that pregnant women deliver their child in proper health setting, where life saving equipment and hygienic condition can also reduce the probability that may cause death or illness to mother and child [44]. Delivery conducted in a medical institution assisted by health professionals is termed as Institutional delivery and Deliveries that take place in home is termed as home deliveries [32], again the institutional delivery is classified in two categories: public and private institution. In this survey, place of delivery were classified into 17 categories, however, for the purpose of this study all the public and government sectors institution have been combined under public institution; and the private sector and NGO related institution are recoded into private institution. The study considered postnatal care check-up within 42 days after child birth, is a critical maternal healthcare service indicator [39]. The postpartum period is crucial for women, as during this period they may develop serious, life-threatening complications after delivery. Postpartum haemorrhage being an important cause of maternal deaths because a considerable proportion of maternal deaths occur during this period [45]. A postnatal care visit is an ideal time to educate a new mother on how to care for herself and her newborn [46]. Therefore, it is highly recommended that women receive at least three postnatal checkups, the first within 24 hours of delivery, the second on the third day following delivery, and the third on the seventh day after delivery [33].

### Defining Predictor Variables

The predictor variable for this study is Birth Preparedness. In an effort to prevent unnecessary delays related to delivery care, the Ministry of Health and Population [MOHP] has implemented the birth preparedness package, which outlines steps that mothers should take to prepare for their birth [47]. Adherence to these guidelines reduces delays in accessing delivery services, which can save lives, especially among women living in rural locations [48]. The guidelines recommend that families should save money for emergencies, arrange transportation in advance based on local conditions, identify persons who can and are eligible to donate blood if required, identify and contact health facilities and health workers who can provide services, and have a clean delivery kit [49].

This study used Principle Component Analyses (PCA) to construct a summary indicator of birth preparedness by using various components suggested by MOHP. Birth preparedness levels were based on eight tools; each tools were assigned a weight (factor score) generated through principal component analysis, and the resulting asset scores standardized in relation to a normal distribution with the mean of zero and standard deviation of one. The score distribution was used to determine poor preparation, moderate preparation and well preparation, and then no preparation category is also added into three categories and computed a new variable— scale is varying from ‘no’ to ‘well preparedness’ in birth preparation. The resultant birth preparedness index was used to examine the levels of birth preparedness among women.

This study has also considered socioeconomic and demographic predictors as background characteristic of the women. Socioeconomic characteristics like Ecological zone, Place of residence, women's education, household wealth quintile and women's autonomy index. Demographic characteristics include age of women, and pregnancy complication.

Household wealth quintile: Due to reporting bias in income, which is a direct economic status variable, we have used proxy economic indicator that is wealth index. It is a composite index constructed based on household assets. We have taken into account the rural urban differences in nature of assets for construction of wealth quintile. All the variables in construction of wealth index are dichotomized (0 and 1) into household possesing the asset or not (yes/no). Principle Component Analyses (PCA) has been used to produce the common score for categorical and continuous variable for each household. Separate factor scores are produced for a household in urban area and rural area using area-specific indicators. The third step combines the separate area- specific factors score to produce a nationally applicable combine wealth index by adjusting area-specific score through a regression on common factors scores and the resulting assets scores are standardized in relation to the normal distribution with the mean of zero and standard deviation of one. Further, the sample is divided into five equal quintiles: Poorest, Poorer, Middle, Richer and Richest.

The similar procedure which adopted in construction of household wealth quintile is also adapted in construction of women autonomy index. It is a composite index constructed, based on seven variables related to women's freedom of decision making in case of their own personal care, health care, household assets and other daily needs purchase and freedom of mobility.

### Analytical Approach

All the statistical analyses of this study were done by using SPSS version 20 and Microsoft excel Program. The analyses of this study were carried out in two stages: in the first stage, the univariate and bivariate models were used to estimate levels of birth preparedness and variation in birth preparedness levels among the women of different socioeconomic characteristic. The second stage involves the association of birth preparedness with place of delivery and related care is also examined. Adjusted effects of birth preparedness on the place of delivery and related care were estimated after controlling socioeconomic background characteristics of women by applying binary logistic regression.

### Ethical Statement

Ethical approval for this survey was provided by an independent expert committee of the Population Division, Ministry of Health and Population, Government of Nepal and Macro-International Maryland, USA. A written informed consent has been obtained from all the respondents or next-of-kin when respondents were under 18 years of age, in the age group 15–59 before conducting interviews. The information collected in the survey was purely used for research works and the name and place of the respondents has remained anonymous [43].

## Results

**Figure 1:**
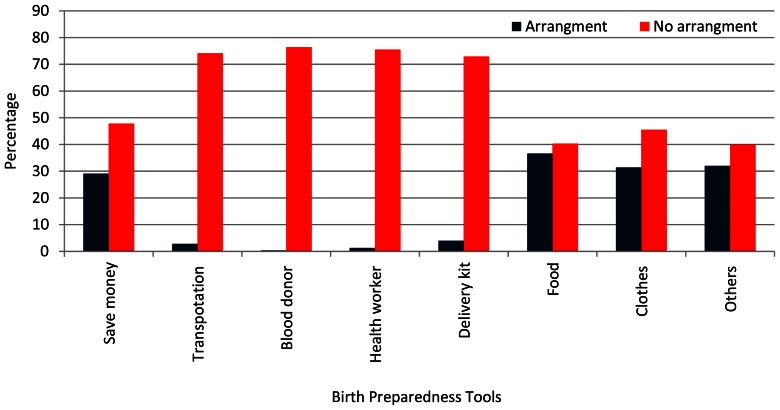
Percentage distribution of women by arrangement (Yes/No) of different birth preparedness tools, Nepal, 2011.

**Figure 2:**
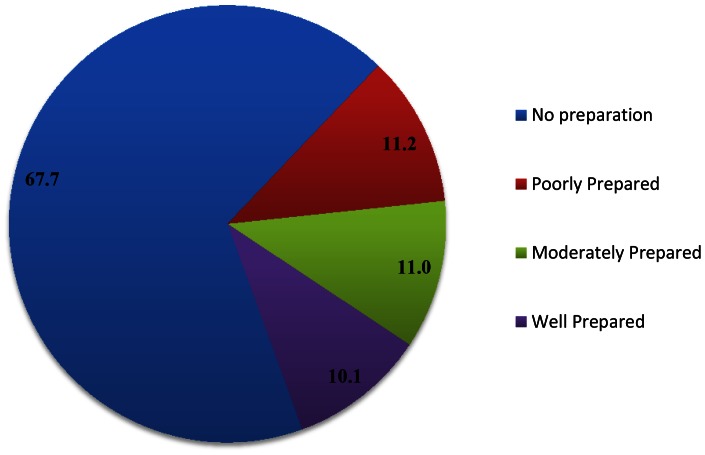
Composition of women by birth preparedness levels in Nepal, 2011.

Figure 1 shows the percentage distribution of women in the categories of birth preparation for each tool of birth preparation considered. The NDHS-4 considered seven tools of birth preparedness: whether respondent saved money or not, could arrange transportation or not, arranged blood donor or not, contacted health worker or not, arranged delivery kit or not, arranged food or not, and arranged clothes or not. The results reveal that only 30 percent of Nepali women saved money for delivery care and related emergency. However, just five percent pregnant women could arrange transportation for the emergency care. Similarly, less than 5 percent women contacted with health worker and arranged blood donor, if in case they need it for emergency. Around five percent of pregnant women arranged delivery kit for safe delivery. By using seven tools of the birth preparedness described in Figure 1, a birth preparedness index is constructed to assess the level of birth preparedness. Figure 2 presents pie diagram showing the composition of women by different levels [no preparation, poor preparation, moderate preparation, well preparation] of birth preparedness. The results reveal that around 70 per cent women in Nepal not have any preparation while only 10 per cent women were well prepared for delivery.

Table 1 shows the level of birth preparedness among Nepali women by demographic and socioeconomic characteristics. By pregnancy complication status, the results indicate that the women with pregnancy complications are more prepared compared to other categories; it is also expected that women with pregnancy complications need more preparation for delivery compared to their counterparts. The birth preparedness among women in lower and higher age band is less compared to women in middle age groups. The lower and higher age bands is considered to be risky age groups for delivery, however, women of these age groups showed poor birth preparedness. By the ecological zones, the proportions of women with no birth preparedness were found more in Mountain (72 %) than Hill (69 %) or Terrain zones (66 %). There is a huge urban-rural gap in birth preparedness. The proportion of women with no birth preparation in rural areas is 40 percent higher compared to urban areas. Education of women shows greater association with birth preparedness. The proportions of women who do not have any birth preparedness among no education category (77%) are nearly three times greater compared to women in educated category (28 %). The results indicate huge variation in birth preparedness by wealth quintile. The proportion of women with no birth preparedness is almost double in poorest wealth quintile (80%) as compared to richest wealth quintile (45%). However, women autonomy also emerges as a major contributor of birth preparedness among Nepal women. The women with well birth preparedness are three times greater among women of higher autonomy compared to lower autonomy.

**Table 1:** Levels of Birth Preparedness by background characteristics, Nepal, 2011.

Background Characteristics of women	Birth Preparedness				N
	Poor Preparation	Moderate Prepared	Well Prepared	Chi- Square	
**Pregnancy Complication**					
Yes	32.20	33.10	34.60	1.35*	524
No	30.00	30.00	40.00		106
**Age of women**					
15–24	40.90	33.00	26.10	14.37***	284
25–34	31.90	35.10	33.00		483
35+	24.00	30.80	45.20		85
**Ecological Zone**					
Mountain	46.00	32.00	22.00	10.44**	127
Hill	31.00	35.90	33.10		298
Terni	34.30	33.20	32.50		427
**Place of residence**					
Rural	37.7	31.9	30.5	4.82*	571
Urban	30.6	38.1	31.3		281
**Education**					
No education	38.20	34.50	27.30	4.15	318
Primary and Secondary	32.60	32.10	35.20		409
Higher	24.80	38.80	36.40		125
**Wealth Quintile**					
Poorest	27.6	35.5	36.8	28.70***	152
Poorer	35..4	37.2	27.4		164
Middle	51.4	22.5	26.1		142
Richer	36.7	30.9	32.4		188
Richest	28.6	40.80	30.6		206
**Women Autonomy Index**					
Low	100.00	**	**	1507.76***	301
Medium		100.0			253
High		14.20	85.80		298

Significance Levels- p<0.10*, p<0.05**, p<.01***.

Table 2 presents the percentage of women delivered a baby in a health facility, type of health facility and check up after delivery by status in various birth preparedness components and overall birth preparedness levels. The results clearly indicate that out of those women, who have saved money for delivery turn-up in greater proportion to institutional delivery (56%), where among those who have not saved money only less than 30 percent women delivered in a health facility without any prior strategy. However, results of type of health facility (Private/Public) for delivery indicate that irrespective of whether they saved money or not, greater proportion of women delivered in public health facility (75%) compared to private health facility (25%). Among those women, who arranged for transportation are heavily inclined towards institutional delivery (84%), whereas among those women who have not arranged transportation only 37 percent of them went for institutional delivery. Lower than normal number of healthy red blood cells in the body of pregnant women demand blood donor during or at the time of delivery. Among those women who have arranged blood donor greatly turn-up to institutional delivery (84%). Unlike in other birth preparedness categories, the proportions of women who have arranged blood donor are equally going to government and private hospitals. The other birth preparedness categories like arranging for safe delivery kit, food and clothes and other required material are showing poor association with the place of delivery. Overall, among the women who have well prepared for delivery and turn-up to institutional delivery are two times higher compared to those who have poor preparation and turn-up to institutional delivery. However, on an average 70 per cent of institutional deliveries are in public health facility.

**Table 2:** Institutional delivery, type of health facility for delivery and related care by schemes of birth preparedness, Nepal, 2011.

Birth preparedness tools	Institutional delivery	Type of health facility for delivery		Check up after delivery	N
		Private	Public	Yes	Total
**Saved money**					
Yes	55.60	74.80	25.20	61.20	1543
No	28.90	72.50	27.50	38.80	2534
**Arranging for transport**					
Yes	83.70	70.90	29.10	81.00	147
No	36.90	73.90	26.10	19.00	3930
**Found blood donor**					
Yes	84.20	56.20	43.80	78.30	23
No	38.30	73.90	26.10	21.70	4054
**Contacted health worker**					
Yes	51.40	75.70	24.30	74.60	71
No	38.20	73.60	26.40	25.40	4006
**Bought safe kit**					
Yes	21.50	77.50	22.50	43.90	212
No	39.30	73.60	26.40	56.10	4006
**Arrange food**					
Yes	37.90	74.20	25.80	43.20	1939
No	38.90	73.40	26.60	56.80	2138
**Arrange clothes**					
Yes	50.10	73.90	26.10	55.30	1663
No	31.20	73.60	26.40	44.70	2414
**Others**					
Yes	31.10	85.70	14.30	38.10	63
No	38.60	73.50	26.50	61.90	4014
**Birth preparedness index**					
No preparation	28.50	7.20	21.3	32.50	1300
Poor	42.40	12.3	30.00	53.80	237
Moderate	46.80	11.3	35.30	55.50	231
Well	51.80	8.60	40.50	60.20	204

Table 3 presents the logistic regression estimates (Odds Ratios [OR]) of institutional delivery by birth preparedness after controlling all other relevant background characteristics. The adjusted effects of birth preparedness on institutional delivery indicate a similar pattern as followed in bivariate results. The results show that, after controlling all relevant socioeconomic predictor, those women with birth preparedness (OR = 1.412, <0.05) show greater likelihood of going for institutional delivery compared to those who do not have birth preparedness (OR = 1). Wealth status shows a clear hierarchical pattern: increasing wealth status is associated with the increase in institutional delivery. Similarly, mother's education and women autonomy also show a greater association with the institutional delivery.

**Table 3:** Odds ratios showing the relationship of birth preparedness on institutional delivery and other background characteristic - Result of logistic analysis.

Factors	Exp(B)	95% C.I. for EXP(B)	
		Lower	Upper
**Birth preparedness levels**			
No Preparation	1.00		
Poor Preparation	2.185***	1.648	2.898
Moderate prepared	2.752***	2.069	3.662
Well prepared	3.133***	2.318	4.234
**Women age**			
15–24	1.00		
25–34	0.618**	0.429	0.892
35+	0.603*	0.32	1.134
**Place of residence**			
Urban	1.00		
Rural	1.026	0.614	1.713
**Region**			
Mountain	1.00		
Hill	0.808*	0.495	1.321
Terai	0.512***	0.351	0.747
**Mother education**			
No education	1.00		
Primary & Secondary	1.899	1.294	2.786
Higher education	5.178*	2.763	9.705
**Wealth quintile**			
Poorest	1.00		
Poorer	1.223**	0.699	2.138
Middle	1.541***	0.869	2.731
Richer	4.314***	2.529	7.358
Richest	11.327***	6.059	21.177
**Women autonomy**			
Low	1.00		
Medium	1.298	0.927	1.816
High	1.351*	0.979	1.864
**Constant**	0.414**		

Significance levels- p<0.10*, p<0.05**, p<.01***.

Table 4 presents the adjusted effects of birth preparedness on check-up after delivery has been estimated by logistic regression. This model also includes levels of birth preparedness as main predictors of check up after delivery, but the model is controlled for other relevant socio economic variables. Result shows that, after controlling the relevant variable in socioeconomic variables, the likelihood of going for check-up after delivery is three times greater among women with well birth preparedness (OR = 3.13, p<0.05) compared to no birth preparedness (OR = 1). Wealth status also emerges as one of the important predictors of visiting health professional after delivery: increasing wealth is associated with greater postnatal check-ups. The education level of women is also positively associated with visit to health check-ups after delivery. Moreover, women autonomy is associated with more and timely check-ups after delivery.

**Table 4:** Odds ratios showing the relationship of birth preparedness on check up after delivery and other background characteristic - Result of logistic analysis.

Factors	Exp(B)	95% C.I. for EXP(B)	
		Lower	Upper
**Birth preparedness levels**			
No Preparation			
Poor Preparation	2.409***	1.747	3.323
Moderate prepared	2.562***	1.876	3.5
Well prepared	3.137***	2.302	4.274
**Women age**			
15–24			
25–34	0.816	0.558	1.192
35+	0.911	0.49	1.697
**Place of residence**			
Urban			
Rural	0.618**	0.419	0.911
**Region**			
Mountain			
Hill	1.083	0.636	1.846
Terai	0.921	0.554	1.533
**Mother education**			
No education			
Primary & Secondary	1.712***	1.146	2.556
Higher education	5.324***	2.7	10.499
**Wealth quintile**			
Poorest			
Poorer	1.12	0.643	1.95
Middle	1.321	0.732	2.353
Richer	3.049***	1.745	5.326
Richest	4.882***	2.609	9.135
**Women autonomy**			
Low			
Medium	1.507	0.555	4.095
High	1.233	0.813	1.87
**Constant**	0.396***		

Significance levels- p<0.10*, p<0.05**, p<.01***.

## Discussion

This study assesses birth preparedness levels among Nepal women and its association with institutional delivery and postnatal check-ups based on the recent data of Nepal demographic and Health Survey. The results of this paper foster that, level of birth preparedness among Nepal women is poor and it varies considerably with socioeconomic factors. Though, the level of birth preparedness is greater among women with pregnancy complications, lower age group, and higher education and economic status and with greater women autonomy but women's age and women autonomy emergences as dominant predictors of birth preparedness as compared to other factors. Moreover, women, who are well prepared for birth, have greater likelihood of going for institutional delivery than their counterparts. However, irrespective of level of birth preparedness, women in Nepal preferred to deliver in public health facility than private health facility. This could be because of the attraction towards monetary incentives for institutional deliveries in government health facilities.

Among all birth preparedness components, saving money, arranging transport, contacting health worker and arranging blood donors become very important factors for delivering a baby in a health facility. The saving money is important especially for women belonging to poor household economic status [7]. Arranging transport is critical for women living in rural areas and away from health facility. Underweight and anemic women must arrange for a blood donor under birth preparedness strategy [44]. However, a notable proportion of women are also going for institutional deliveries without prior preparation, which hints about emergency deliveries. Among other factors, economic and educational status and women autonomy are also positively related with the institutional delivery and check-up after delivery.

Overall, the findings of this study suggest that, the birth preparedness package launched by government of Nepal in the year 2008–2009 [44], yet to reach all sections of women such as poorest of poor, illiterate and women with lower autonomy, living in the hill region. This may be a plausible reason for greater proportions of women in these categories, as they are not turning-up for institutional delivery and check-up after delivery. This asserts that birth preparedness is paying a critical role in institutional delivery and check-up after delivery. At policy perspective, this study reinstates that birth preparedness is important for safe deliveries and bringing women to health facilities for delivery and check-up after delivery. However, a notable proportion of woman does not have birth preparedness, and many of them going to health facilities for deliveries without prior preparation, therefore, the laggard countries like Nepal need to strengthen existing policy interventions to reduce emergency complications and deliveries.

Though, Nepal is promoting safe motherhood through initiatives such as providing financial assistance through maternity incentives schemes to women for institutional deliveries and seeking skilled delivery care in a health facility but such facilities can help women only in terms of curative care rather than preventive care. As observed from the results of this study that a considerable proportion of women going for institutional deliveries with no birth preparedness or with poor preparation, which are mostly emergency and complicated deliveries. In such case, even with monetary incentives the damage cannot be undone. Therefore, it is strongly recommended that Nepal should revise the existing system of monetary benefits to women for institutional deliveries. Women should get benefits, right from antenatal care visits (ANCs) at least in three instalments so that women can come for at least three ANC visits. Moreover, number of earlier studies in a global context [50], [51], [52], [53] also shows that, institutional deliveries without adequate antenatal care visits will not yield greater benefits which are also true for Nepal. Policy makers in Nepal should focus not only on increasing of institutional deliveries but also institutional deliveries with adequate birth preparedness. Birth preparedness helps to reduce the delays that occur when women experience obstetric complications, such as recognizing the complication and deciding to seek care, reaching a facility where skilled care from qualified providers at health facility. Preparedness also helps pregnant women and mothers to acquire skills and confidence needed to make birth a positive experience as it dissolves fears of pregnancy and new born care. Therefore, universal birth preparedness is must for reaching goal 4 and 5 of MDGs.
